# Behavioral Forgetting of Olfactory Learning Is Mediated by Interneuron-Regulated Network Plasticity in *Caenorhabditis elegans*

**DOI:** 10.1523/ENEURO.0084-22.2022

**Published:** 2022-08-26

**Authors:** Jamine Hooi-Min Teo, Itsuki Kurokawa, Yuuki Onishi, Noriko Sato, Tomohiro Kitazono, Terumasa Tokunaga, Manabi Fujiwara, Takeshi Ishihara

**Affiliations:** 1 Graduate School of System Life Science, Kyushu University, Fukuoka 819-0395, Japan; 2 Department of Artificial Intelligence, Faculty of Computer Science and Systems Engineering, Kyushu Institute of Technology, Fukuoka 820-8502, Japan; 3 Department of Biology, Faculty of Science, Kyushu University, Fukuoka 819-0395, Japan

**Keywords:** C. elegans, circuit plasticity, forgetting, memory, olfactory learning

## Abstract

Forgetting is important for animals to manage acquired memories to enable adaptation to changing environments; however, the neural network in mechanisms of forgetting is not fully understood. To understand the mechanisms underlying forgetting, we examined olfactory adaptation, a form of associative learning, in *Caenorhabditis elegans*. The forgetting of diacetyl olfactory adaptation in *C. elegans* is regulated by secreted signals from AWC sensory neurons via the TIR-1/JNK-1 pathway. These signals cause a decline of the sensory memory trace in AWA neurons, where diacetyl is mainly sensed. To further understand the neural network that regulates this forgetting, we investigated the function of interneurons downstream of AWA and AWC neurons. We found that a pair of interneurons, AIA, is indispensable for the proper regulation of behavioral forgetting of diacetyl olfactory adaptation. Loss or inactivation of AIA caused the impairment of the chemotaxis recovery after adaptation without causing severe chemotaxis defects in the naive animal. AWA Ca^2+^ imaging analyses suggested that loss or inactivation of AIA interneurons did not affect the decline of the sensory memory trace after the recovery. Furthermore, AIA responses to diacetyl were observed in naive animals and after the recovery, but not just after the conditioning, suggesting that AIA responses after the recovery are required for the chemotaxis to diacetyl. We propose that the functional neuronal circuit for attractive chemotaxis to diacetyl is changed temporally at the recovery phase so that AIA interneurons are required for chemotaxis, although AIAs are dispensable for attractive chemotaxis to diacetyl in naive animals.

## Significance Statement

Forgetting is important to enable animals to adapt to changing environments; however, the mechanisms of forgetting are poorly understood at the molecular and cellular levels. In this study, we found that a pair of interneurons in the olfactory circuit of *Caenorhabditis elegans* are indispensable for behavioral forgetting, but not for regulation of the sensory memory trace, in simple olfactory learning. These findings suggest that neuronal circuits are important for regulating forgetting by managing memory and also for the generation of appropriate behavioral responses.

## Introduction

Animals are able to learn and form memories, depending on the experience they gain from their surroundings; however, to adapt to changing environments, it is essential that dispensable information is discarded to manage accumulating memories. Recent studies reveal that memories can be actively forgotten by interference with other memories or by activating forgetting in neurons that are important for maintaining memories (Hardt et al., 2013; Davis and Zhong, 2017). However, the molecular mechanisms and neural networks engaged in forgetting are not well understood.

The complexity of brain structure in higher organisms makes studies on active forgetting at molecular and cellular levels challenging; therefore, invertebrates with simple nervous systems, such as *Caenorhabditis elegans*, have been used (Inoue et al., 2013; Hadziselimovic et al., 2014). Despite a simple neural network, *C. elegans* shows behavioral plasticity toward various stimuli, such as volatile and water-soluble chemicals (Bargmann et al., 1993; Colbert and Bargmann, 1995; Saeki et al., 2001). In the well studied neural network of *C. elegans* (White et al., 1986; Cook et al., 2019), most attractive volatile odorants, such as diacetyl and isoamyl alcohol, are sensed by two pairs of amphid sensory neurons, AWA and AWC, respectively, and these neurons have distinctive sensory mechanisms (Bargmann et al., 1993; Sengupta et al., 1994, 1996; Colbert and Bargmann, 1995; Colbert et al., 1997; L’Etoile and Bargmann, 2000; Bargmann, 2006). These amphid sensory neurons make synapses to first-layer interneurons, mainly AIA, AIB, and AIY, which also regulate the plasticity of various behaviors, such as associative learning (Rankin et al., 1990; Tomioka et al., 2006; Chalasani et al., 2010; Cho et al., 2016), as well as integrate multiple sensory signals, including contradicting information (Shinkai et al., 2011; Larsch et al., 2015; Dobosiewicz et al., 2019; Wolfe et al., 2019), to generate appropriate cellular responses and animal behavior.

In invertebrates, despite their simple neural networks, several studies showed that forgetting is actively regulated. In *Drosophila*, dopamine neurons regulate both learning and active forgetting through distinctive dopamine receptors in mushroom body neurons (Berry et al., 2018, 2012). One of the dopamine receptors in mushroom body neurons, dDA1, leads to memory formation (Berry et al., 2012), while for forgetting, another receptor, DAMB, activates Scribble scaffold to initiate forgetting by actin cytoskeleton remodeling (Shuai et al., 2010; Cervantes-Sandoval et al., 2016; Berry et al., 2018, 2015, 2012). *C. elegans* is also used to study active forgetting through olfactory adaptation, a form of associative learning (Colbert and Bargmann, 1995; Bargmann, 2006).The forgetting of diacetyl olfactory adaptation, which is sensed by AWA olfactory neurons, is regulated by the TIR-1/JNK-1 pathway in another type of olfactory neuron, AWC. In wild-type and mutant animals, which are defective in the TIR-1/JNK-1 pathway, the sensory Ca^2+^ response of AWA neurons to diacetyl is positively correlated with behavioral change through memory formation and forgetting, suggesting that the sensory response can be considered as the sensory memory trace and that the memory trace in AWAs is actively and non-cell-autonomously regulated by AWCs (Inoue et al., 2013; Kitazono et al., 2017). In addition, a membrane protein, MACO-1, and a tyrosine kinase pathway, SCD-2/HEN-1, regulate the forgetting (Kitazono et al., 2017). Another study showed that, similar to Rac1 in *Drosophila*, the Arp2/3 complex, which regulates the actin cytoskeleton in AVA interneurons, is important for forgetting downstream of the RNA binding protein *Musashi* (Hadziselimovic et al., 2014). Although these studies indicate that active forgetting is important, even in simple learning paradigms of model organisms, the corresponding neural network has not been fully revealed.

Here, we demonstrate that a pair of neurons in *C. elegans*, AIA interneurons, which are the first-layer interneurons in olfactory circuits, is required to regulate forgetting processes of olfactory adaptation. Although absence or inactivation of functional AIA interneurons (AIA^–^) slightly affect attractive chemotaxis to diacetyl, it caused prolonged retention of the olfactory adaptation to diacetyl, suggesting that AIAs accelerate forgetting. Calcium imaging analyses showed that, although the behavioral response in AIA^–^ animals did not recover after cultivation for 4 h, the calcium responses to diacetyl in AWA animals were recovered. These results suggest that AIAs are indispensable for the behavioral response of the olfactory adaptation forgetting mechanism, probably because the functional neuronal circuit is changed temporally so that AIAs are required for the chemotaxis.

## Materials and Methods

### Strains and culture

All strains were cultured on nematode growth medium (NGM) agar plates seeded with *Escherichia coli* strain OP50 (Brenner, 1974) and were grown at 20°C before experiments. In all experiments, we used young adult hermaphrodites prepared as described in each section (Table 1).

**Table 1:** Strain list

Strain name	Genotype	Source
	N2	CGC
	*tir-1(tm3036)*	National Bioresource Project
RB1085	*tir-1(ok1052)*	CGC
JN578	*peIs578[npr-9p::casp1, npr-9p::venus, unc-122p::mCherry]* (AIB-)	Satoh et al., 2014
JN579	*peIs579[ttx-3p::casp1, ttx-3p::venus, lin-44p::gfp]* (AIY-)	Satoh et al., 2014
JN580	*peIs580[ins-1(short)p::casp1, ins-1(short)p::venus, unc-122p::gfp]* (AIA^–^)	Satoh et al., 2014
QD155	*qjEx3*[*gcy-28.dp::mec-4(d), gcy-28::gfp, lin-44p::gfp*]	Shinkai et al., 2011
QD156	*qjEx4*[*gcy-28.dp::unc-103(gf), myo-3p::gfp*]	Shinkai et al., 2011
QD139	*lin-15(n765ts)*; *qjEx39*[*odr-10p::YC3.60*, pBLH98]	Inoue et al., 2013
QD140	*tir-1(tm3036)*; *lin-15(n765ts)*; *qjEx39* [*odr-10p::YC3.60*, pBLH98]	Inoue et al., 2013
QD157	*peIs578[npr-9p::casp1, npr-9p::venus, unc-122p::mCherry]*; *peIs579[ttx-3p::casp1, ttx-3p::venus, lin-44p::gfp]* (AIB-; AIY-)	This article
QD165	*tir-1(ok1052)*; *qjEx4* [*gcy-28.dp::unc-103(gf), myo-3p::gfp*]	This article
QD164	*tir-1(tm3036)*; *qjEx3* [*gcy-28.dp::mec-4(d), gcy-28::gfp, lin-44p::gfp*]	This article
QD166	*tir-1(tm3036); qjEx4*[*gcy-28.dp::unc-103(gf), myo-3p::gfp*]	This article
QD153	*lin-15(n765ts)*; *qjEx39*[*odr-10p::YC3.60*, pBLH98]; *peIs580[ins-1(short)p::casp1,* *ins-1(short)p::venus, unc-122p::gfp]*]	This article
QD272	*qjEx52*[*gcy-28.dp*::GCaMP6f, *gcy-28.dp*::paQuasAr3-citrine, *lin-44p::gfp*]	This article

CGC, *Caenorhabditis* Genetics Center.

### Behavioral assay

Chemotaxis toward attractive odorants was performed on assay plates (2% Bacto agar, 50 mm NaCl, 10 mm K_2_HPO_4_, pH 6, 1 mm MgSO_4_, 1 mm CaCl_2_) with 1:100 dilutions of odorants (diacetyl and isoamyl alcohol; Bargmann et al., 1993). During behavioral assays, animals were placed in the middle of the assay plate while the odorant and control solution (ethanol, the odorant diluent) were spotted on opposite sides of the plate. The chemotaxis index was calculated as (*A* – *B*)/*N*, where *A* refers to the number of animals within 1.5 cm of the odorant spot, *B* refers to the number of animals within 1.5 cm of the control spot, and *N* is the total number of animals. In the forgetting assay (Inoue et al., 2013), adult animals were first washed three times with S-basal buffer (100 mm NaCl, 50 mm K_2_HPO_4_, pH 6, 0.02% gelatin; naive) and pre-exposed to 1:5000 diluted diacetyl or isoamyl alcohol in S-basal buffer with slow rotation for 90 min at room temperature (adaptation). Next, the worms were washed once and allowed to recover on OP50-seeded NGM plates for 4 h (recovery). In the extended forgetting assay, animals were recovered on OP50-seeded NGM plates for 24 h, and behavioral assays after recovery were conducted after 4, 8, and 24 h of recovery.

### Calcium imaging

Calcium imaging of AWA neuron responses toward diacetyl was performed using AWA-cameleon YC3.60-expressing animals (Inoue et al., 2013). The day before imaging, 30–45 animals (L4 - young adults) were picked and cultured at 20°C. A 1:10^−7^ dilution of diacetyl was used for odor stimulation, and a 1:10^−3^ dilution of diacetyl was used for adaptation. Adaptation and recovery were conducted as described for the behavioral assay. During Ca^2+^ imaging, odor stimulation was applied to the animal for 60 s (30th to 90th second of recording). Fluorescence images of AWA sensory neurons were acquired using a microscope (model BX53-FL, Olympus) equipped with a 60× objective lens (UPLSAPO 60XW, Olympus) and a dual CCD camera (model ORDA-D2, Hamamatsu). Cameleon YC3.60 was excited using X-Cite 120 fluorescence lamp illuminators (EXFO). The fluorescence ratio of yellow fluorescent protein (YFP) to cyan fluorescent protein (CFP) in Cameleon YC3.60 was analyzed using an AQUACOSMOS system (version 2.60; Hamamatsu). The calculation (*R*_max_ – *R*_0_)/*R*_0_ was performed as the peak amplitude of the YFP/CFP ratio during the first 10 s interval after stimulation (*R*_max_) relative to the mean basal ratio (*R*_0_) during the 10 s interval before stimulation. The relative Ca^2+^ response was evaluated by normalized (*R*_max_ – *R*_0_)/*R*_0_ with respect to the average naive value.

For calcium imaging on AIA neurons, we used animals expressing GCaMP6f in AIA neurons by *gcy-28.d* promoter. Animals were cultivated as for AWA imaging. A 1:10^−7^ dilution of diacetyl was used for odor stimulation, and a 1:10^−3^ diacetyl was used for adaptation. Fluorescent images were acquired using a microscope (model BX53-FL, Olympus) equipped with a 60× objective lens (UPLSAPO60XS2, Olympus) and an ORCA-Flash camera with extended focus device of Gemini-2c (Hamamatsu). GCaMP6f was excited using a 470 nm laser (LDI, 89 North) with a dichroic mirror (488/543/635, Semrock) and fluorescence images were captured at 50 ms through an emission filter (512/25, Semrock). The neurite of AIA neurons was analyzed. The fluorescent intensities were normalized by the average response (*R*_0_) of a 5 s time period prior the stimulation.

### Experimental design and statistical analyses

For all experiments, adult hermaphrodites were used. In the behavioral assays, the stage of animals was synchronized by removing adult animals from OP50-seeded NGM plates 16–20 h after transfer (Ishihara et al., 2002). Injection markers, such as *myo-3p::gfp*, *lin-44p::gfp*, *unc-122p::mCherry*, and *unc-122p::gfp*, were used to distinguish transgenic animals with extrachromosomal transgenes, and the values of animals with extrachromosomal transgenes were compared with those of animals without transgenes on the same plates as internal controls.

All values are presented as either the mean ± SEM in a line graph or box plot. Data analyses were performed using Bell Curve for Excel (version 3.22; Social Survey Research Information Co., Ltd.). Statistical significance between means was determined by Student’s *t* test or two-way ANOVA followed by a *post hoc t* test with Bonferroni’s correction. Sample sizes and statistical values are noted in the figure legends (Table 2).

## Results

### AIA interneurons are required to regulate forgetting in AWA olfactory adaptation

*C. elegans* shows strong attractive chemotaxis to diacetyl, which is mainly sensed by AWA sensory neurons. After animals are exposed to diacetyl without food for 90 min, they show significantly weaker responses to diacetyl (olfactory adaptation; Colbert and Bargmann, 1995). The conditioned animals are able to recover the attractive chemotaxis toward diacetyl to a level similar to that of naive animals after cultivation with food for 4 h (recovery), and we consider this recovery as forgetting (Inoue et al., 2013). Consistent with the behavioral change, the Ca^2+^ responses to diacetyl in AWA neurons are decreased after conditioning and recover with cultivation. This correlation between behavior and sensory responses is also observed in mutants defective in the TIR-1/JNK-1 pathway, which function in AWC sensory neurons. In the *tir-1* (*tm3036*)-null mutant, naive animals show the sensory response to diacetyl and, after conditioning, the Ca^2+^ responses decrease to levels similar to those of wild-type animals. However, in *tir-1*-null animals, similar to the behavioral changes, the Ca^2+^ responses in AWAs to diacetyl do not recover with cultivation. Therefore, the forgetting of diacetyl olfactory adaptation in AWA neurons is regulated by AWC sensory neurons via the TIR-1/JNK-1 pathway (Inoue et al., 2013). AWC neurons do not make direct connections to AWAs; therefore, other neurons may be involved in this regulation.

**Figure 1.**
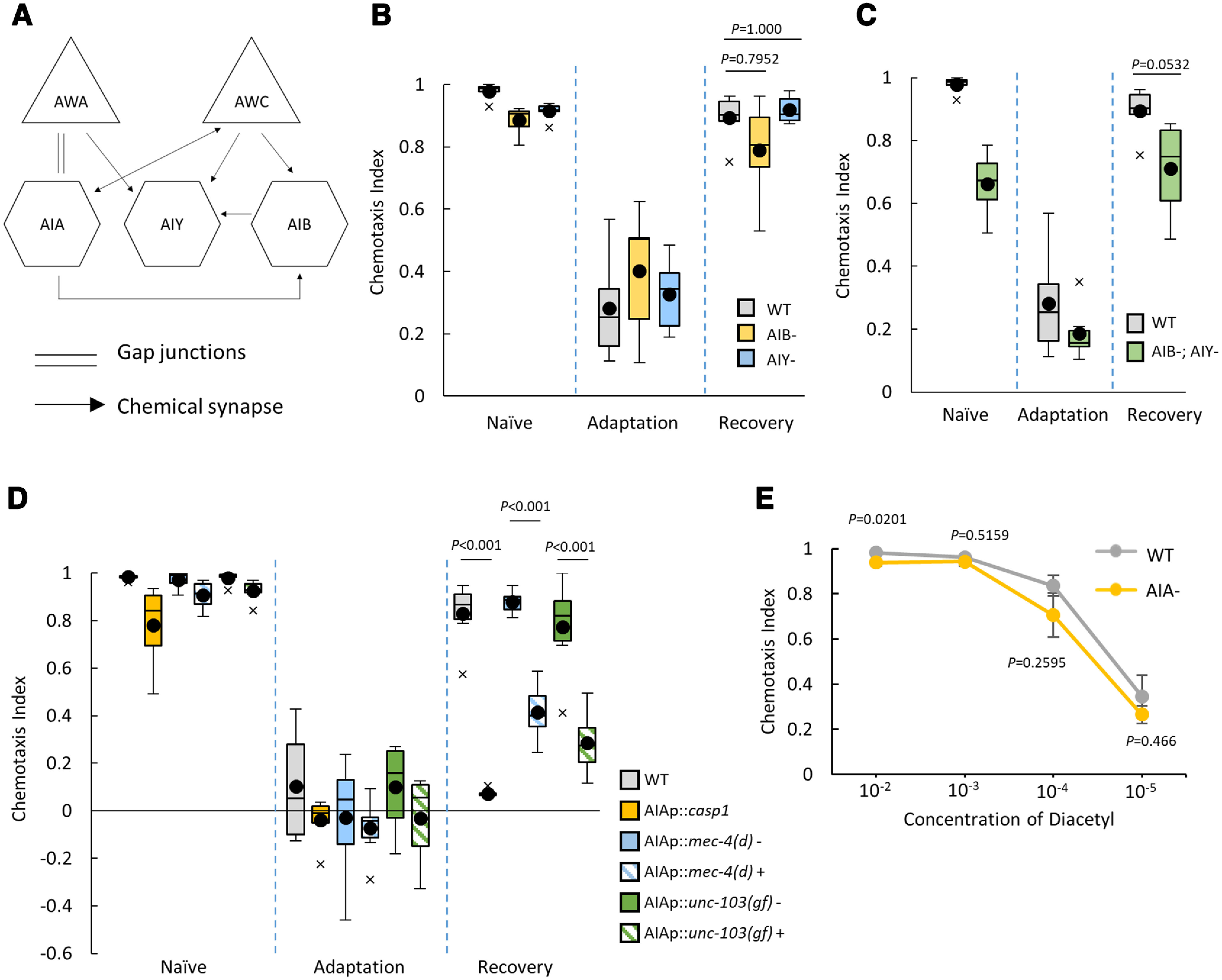
AIA interneurons are required to regulate forgetting of diacetyl olfactory adaptation. ***A***, A simplified neural network for olfactory sensing in *C. elegans* (White et al., 1986; Chalasani et al., 2010; Larsch et al., 2015; Dobosiewicz et al., 2019). ***B–D***, Behavioral assays of animals with ablation of AIA, AIB, and AIY. Chemotaxis of naive, adapted, and 4 h recovered animals was analyzed. Boxes, First to third quartiles (Q1 25th to Q3 75th percentile) of each dataset; black line in the boxes, medians; black dots, mean; whiskers, minimum and maximum, excluding outliers (beyond 1.5-fold interquartile range from Q3 and Q1); x, outliers (AIB^–^, AIY^–^, and AIB^–^; AIY^–^: *n *=* *6, two-way ANOVA, *F*_strain(3,60)_ = 12.11, *p *=* *2.66*e*^−6^, η^2^ = 0.3772; AIA^–^: *n *=* *6, two-way ANOVA, *F*_strain(5,90)_ **=* *21.07, *p *=* *6.86*e*^−14^, η^2^* *=* *0.5393)^a,b^. ***E***, Dose dependency of chemotaxis to diacetyl in AIA^–^ animals (1:10^−2^ diacetyl *n *=* *6, *t*_(5)_ **=* *3.3608, *p *=* *0.0201; 1:10^−3^ diacetyl *t*_(5)_ **=* *0.6986, *p *=* *0.5159; 1:10^−4^ diacetyl *t*_(5)_ **= 1.2714, *p *=* *0.2595; 1:10^−5^ diacetyl *t*_(5)_ **=* *0.7888, *p *=* *0.466; mean ± SEM)^c^. ***B–D***, *Post hoc t* test with Bonferroni’s correction; ***E***, Student’s *t* test. Error bars represent the SEM.

Figure 1*A* shows the olfactory circuit including olfactory sensory neurons, AWAs, and AWCs, and their downstream interneurons (White et al., 1986; Chalasani et al., 2010, 2007; Cook et al., 2019; Dobosiewicz et al., 2019). As shown in Figure 1*A*, AWCs and AWAs mainly relay signals to the first-layer interneurons AIA, AIB, and AIY. Among these, we first examined whether AIB and AIY interneurons, the main synaptic target of AWCs, are involved in forgetting by using animals with genetically ablated AIB and AIY, in which cell-specific cell death is promoted by expressing mouse Caspase 1 (*Casp1*; Satoh et al., 2014). However, in animals without AIBs and/or AIYs, we detected no significant differences in changes of chemotaxis to diacetyl among naive, conditioned, and recovered animals (Fig. 1*B*,*C*). This indicated that AIB and AIY interneurons are dispensable for the regulation of forgetting in this olfactory adaptation.

Next, we examined whether AIAs are important for forgetting using several AIA malfunction strains (AIA^–^). By using an AIA-specific *gcy-28.d* promoter (Shinkai et al., 2011) and an *ins-1* (short) promoter (Satoh et al., 2014), we expressed (1) a hyperactive form of the DEG (degenerin)/epithelial sodium channel MEC-4 [MEC-4(d)] to cause neural toxicity (Harbinder et al., 1997; Shinkai et al., 2011), (2) a constitutively active form of the ERG-like potassium channel UNC-103 [gain of function (gf)] to hyperpolarize and consequently inactive neurotransmission (Shinkai et al., 2011), and (3) *Casp1* for genetic ablation (Satoh et al., 2014). In naive animals, chemotaxis to diacetyl in AIA^–^ strains was weakly defective (Fig. 1*D*,*E*), probably because AIA interneurons are involved in diacetyl perception (Larsch et al., 2015). Despite this weak naive chemotactic defect, we could detect more prominent decreases in chemotaxis to diacetyl after recovery from adaptation in AIA^–^ animals (Fig. 1*D*), indicating that AIA interneurons are required for forgetting diacetyl olfactory adaptation.

### AIA interneurons accelerate forgetting of olfactory adaptation

**Figure 2.**
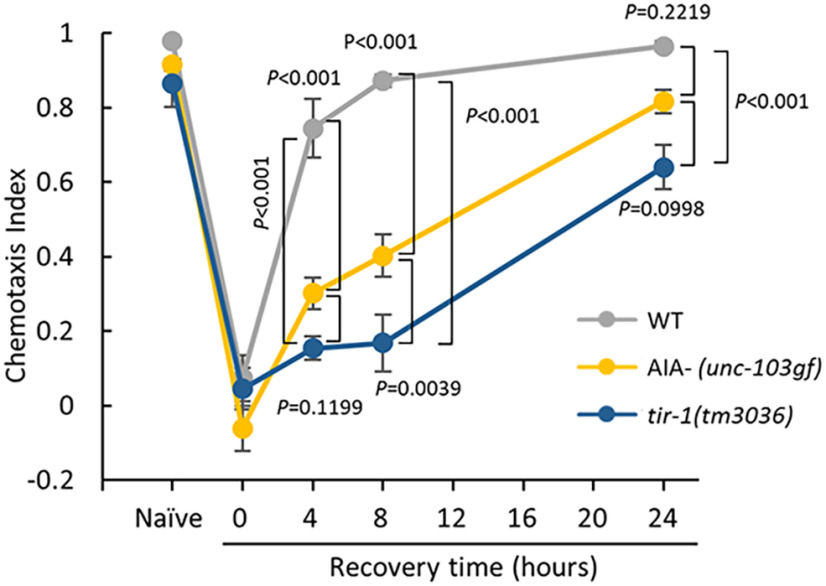
Time course of olfactory adaptation recovery. Time course of chemotaxis recovery after adaptation in *tir-1(tm3036)* and AIA^–^ (*unc-103 gf*) animals. Chemotaxis in naive animals, and after adaptation (0 h), and after 4, 8, and 24 h of recovery was analyzed (*n *≥* *6, two-way ANOVA, *F*_strain(2,9)_ = 61.26, *p *= 4.84*e*^−18^, *η*^2^ = 0.5531; mean ± SEM)^d^. *Post hoc t* test with Bonferroni’s correction. Error bars represent the SEM.

Next, we examined whether AIA^–^ animals completely lost the ability to forget, or decelerated the forgetting progress, as in *tir-1(tm3036)*-null animal (Fig. 2; Inoue et al., 2013). To test this, we analyzed the time course of memory retention for up to 24 h of recovery (4, 8, and 24 h after conditioning; Fig. 2). In the first 4 and 8 h of recovery, although wild-type animals showed full recovery of chemotaxis, AIA^–^ and *tir-1 (tm3036)*-null animals showed very weak recovery. After 24 h, AIA^–^ animals, similar to *tir-1(tm3036)*-null mutants, showed almost full recovery to diacetyl, suggesting that, even without AIAs, animals can slowly forget the memory. AIA interneurons, therefore, accelerate the forgetting of olfactory adaptation.

### AWA neurons recovered their sensory response to diacetyl after adaptation even in the absence of AIAs

**Figure 3.**
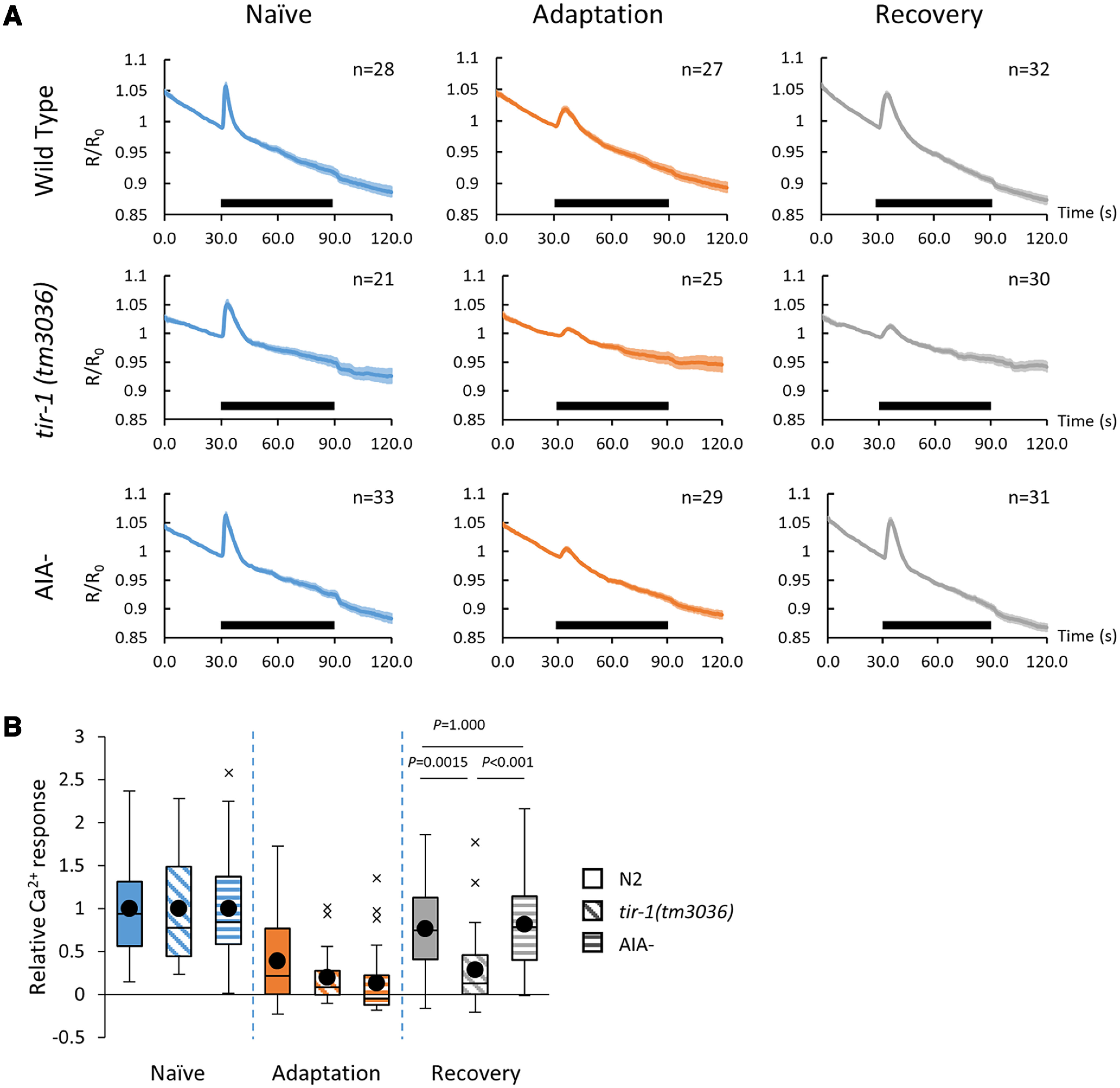
The Ca^2+^ responses to diacetyl of AWA neurons. ***A***, Ca^2+^ responses of AWAs in wild-type, *tir-1(tm3036)*, and AIA^–^ animals in naive, adaptation, and recovery phases (*n* ≥ 21). The black line represents the application of odor stimulation (1:10^−7^ dilution of diacetyl). ***B***, Relative Ca^2+^ responses of AWAs in wild-type, *tir-1(tm3036)*, and AIA^–^ animals. Values are normalized to the average naive value in respective animals. Boxes, First to third quartiles (Q1 25th to Q3 75th percentile) of each dataset; black line in the boxes, medians; black dots, mean; whiskers, minimum and maximum, excluding outliers (beyond 1.5-fold interquartile range from Q3 and Q1); x, outliers (*n*  ≥ 21, two-way ANOVA, *F*_strain(2,247)_ = 3.6626, *p *=* *0.0271, η^2^ = 0.0288; mean ± SEM)^e^. *Post hoc t* test with Bonferroni’s correction. ***A***, Error bars represent the SEM.

The diacetyl-evoked Ca^2+^ response in AWAs is correlated with behavioral change in naive, conditioned, and recovered animals. Therefore, the weakened Ca^2+^ response in AWAs after conditioning can be considered a sensory memory trace. Consistent with this, in *tir-1 (tm3036)*-null mutants, similar to its behavioral response, a weakened Ca^2+^ response in AWAs after conditioning did not recover after 4 h of recovery (Fig. 3; Inoue et al., 2013). To examine whether the ablation of AIAs causes prolonged weakened Ca^2+^ responses in AWAs after conditioning, we analyzed Ca^2+^ responses of AWAs to diacetyl in AIA^–^ animals (naive, adapted, and recovered). In contrast to the behavioral response, the Ca^2+^ response in AIA^–^ animals was recovered after recovery for 4 h (Figs. 1*D*, 3), suggesting that the loss of AIAs decelerates forgetting but not through the inhibition of sensory recovery in AWAs.

### AIA neurons can respond to diacetyl in naive animals and after the recovery

**Figure 4.**
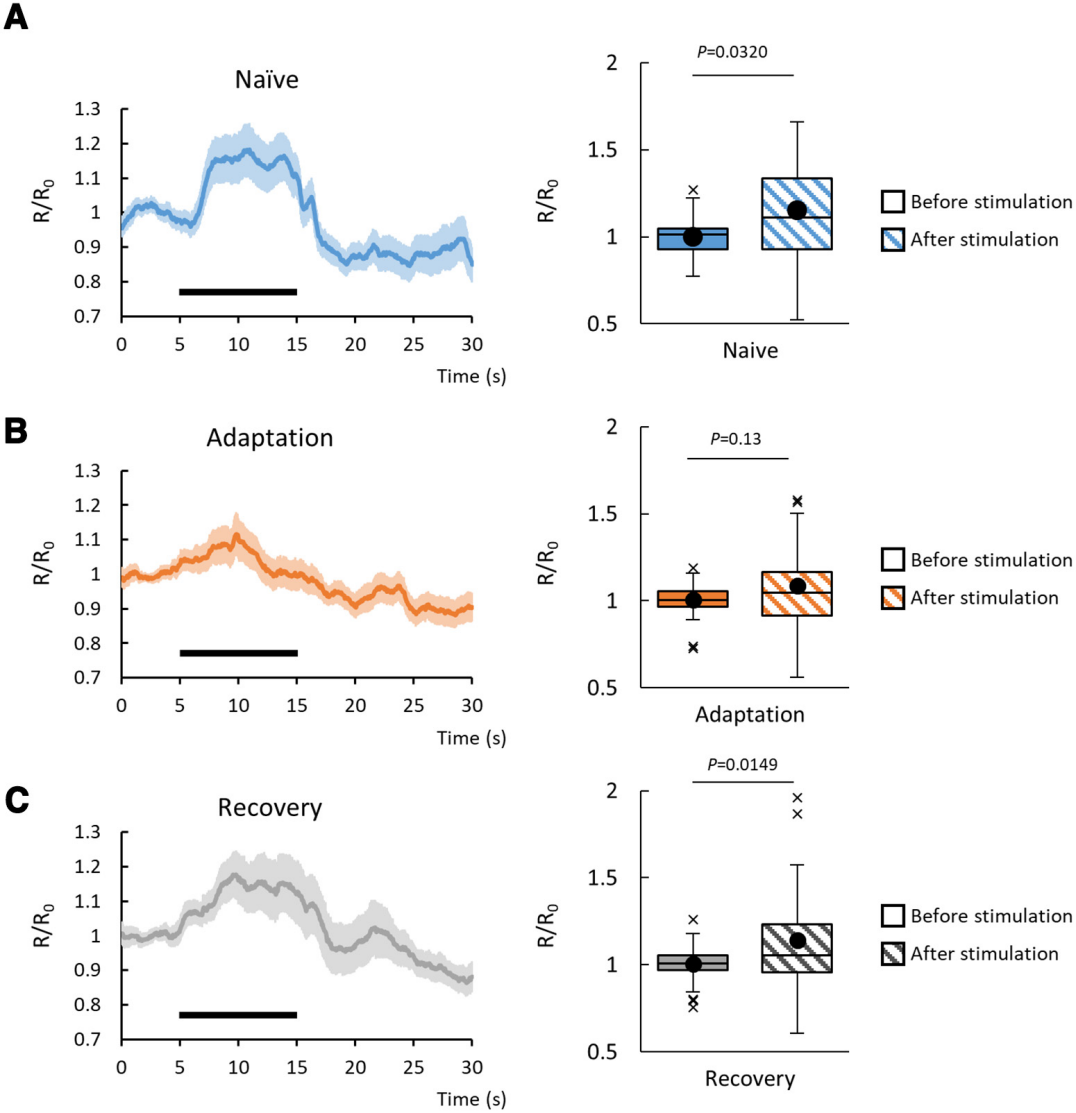
The Ca^2+^ responses to diacetyl of AIA interneurons. ***A–C***, Left, Ca^2+^ responses of AIAs in wild-type animals in naive, adaptation, and recovery phases (*n* = 36; mean ± SEM). Black line represents the application of odor stimulation (1:10^−7^ dilution of diacetyl). Right, Ca^2+^ responses of AIAs before (color; 2.5–5 s) and after (pattern; 7.5–10 s) stimulation in wild-type animals in naive, adaptation, and recovery phases. Boxes, First to third quartiles (Q1 25th to Q3 75th percentile) of each dataset; black line in the boxes, medians; black dots, mean; whiskers, minimum and maximum, excluding outliers (beyond 1.5-fold interquartile range from Q3 and Q1); x, outliers (naive: *n *=* *36, *t*_(35)_ = 2.2336, *p *=* *0.032; adaptation: *t*_(35)_ = 1.5505, *p *=* *0.13; recovery: *t*_(35)_ = 2.5616, *p *=* *0.0149)^f^. Student’s *t* test. ***A–C***, Left, Error bars represent the SEM.

The loss of the functional AIA neurons caused a defect in chemotaxis to diacetyl after the recovery, but not before the conditioning. Recently, Larsch et al. (2015) reported that AIA neurons respond to diacetyl stimulation. Therefore, by using animals expressing GCaMP6f specifically in AIA, we analyzed the responses of AIA neurons to diacetyl in naive animals, immediately after conditioning, and after the recovery. As shown in Figure 4, we found that in naive animals and after the recovery, the fluorescent intensities of AIA neurons responding to the diacetyl stimulation were significantly increased, but such changes were not seen in those immediately after conditioning. This result is consistent with the importance of AIA in the chemotaxis to diacetyl after the recovery.

### AIA interneurons regulate forgetting downstream of the TIR-1 pathway

**Figure 5.**
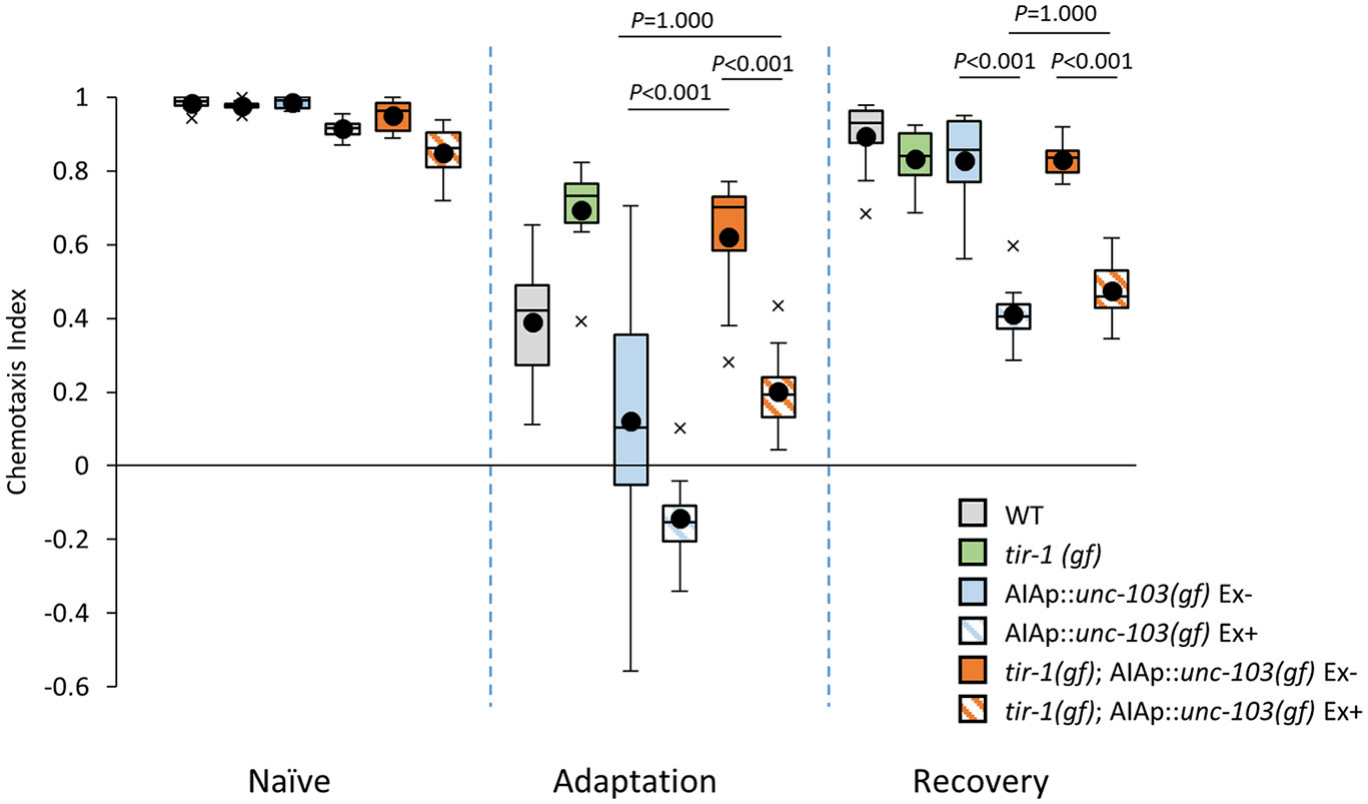
Genetic epistasis between TIR-1 and AIA interneurons. Chemotaxis to diacetyl was analyzed in *tir-1(gf*), AIA^–^, and *tir-1(gf)* animals with no functional AIA in naive, adaptation, and recovery phases. Boxes, first to third quartiles (Q1 25th to Q3 75th percentile) of each dataset; black line in the boxes, medians; black dots, mean; whiskers, minimum and maximum, excluding outliers (beyond 1.5-fold interquartile range from Q3 and Q1); x, outliers (*n *=* *8, two-way ANOVA, *F*_strain(5,126)_ = 38.4268, *p *=* *8.41*e*^−24^, *η*^2^ = 0.6039)^g^. *Post hoc t* test with Bonferroni’s correction.

AIA interneurons are not required to regulate the Ca^2+^ responses in AWAs after recovery; therefore, we suspected that AIA interneurons might regulate forgetting independently of the TIR-1/JNK-1 pathway. We examined the genetic relationship between AIA interneurons and the TIR-1/JNK-1 pathway in the forgetting mechanism, by analyzing genetic epistasis using *tir-1(ok1052 gf)* animals, which show weak adaptation after conditioning probably because of hyperforgetting (Chuang and Bargmann, 2005; Inoue et al., 2013). Consistent with previous studies, *tir-1(ok1052 gf)* animals showed weak adaptation after conditioning (Fig. 5; Inoue et al., 2013). We made *tir-1 (ok1052 gf)* animals without AIA interneurons and found that the animals showed normal adaptation and also prolonged retention of the adaptation (Fig. 5). These phenotypes cannot be distinguished from those of AIA^–^ animals, suggesting that AIA interneurons regulate forgetting downstream of the TIR-1/JNK-1 pathway.

**Table 2:** Statistical table

		Data structure	Type of test	Power (α = 0.05)
a	Figure 1B,C	Normal distribution	Two-way ANOVA	Strains: <0.0001 Conditions: <0.0001
b	Fig. 1D	Normal distribution	Two-way ANOVA	Strains: <0.0001 Conditions: <0.0001
c	Fig. 1E	Normal distribution	Student’s *t* test	1:10^−2^: 0.0201 1:10^−3^: 0.5159 1:10^−4^: 0.2595 1:10^−5^: 0.466
d	Fig. 2	Normal distribution	Two-way ANOVA	Strains: <0.0001 Conditions: <0.0001
e	Fig. 3B	Normal distribution	Two-way ANOVA	Strains: 0.0271 Conditions: <0.0001
f	Fig. 4B	Normal distribution	Student’s *t* test	Naive: 0.032 Adaptation: 0.13 Recovery*:* 0.0149
g	Fig. 5	Normal distribution	Two-way ANOVA	Strains: <0.0001 Conditions: <0.0001
h	Fig. 6	Normal distribution	Two-way ANOVA	Strains: <0.0001 Conditions: <0.0001

## Discussion

Forgetting is important for animals to manage information to properly respond to changing environments. Yet, the neuronal mechanisms for forgetting are not fully understood. In this study, we discovered that a pair of interneurons, AIA interneurons, is required to regulate behavioral forgetting of olfactory adaptation.

### Interneurons accelerate forgetting of olfactory adaptation

**Figure 6.**
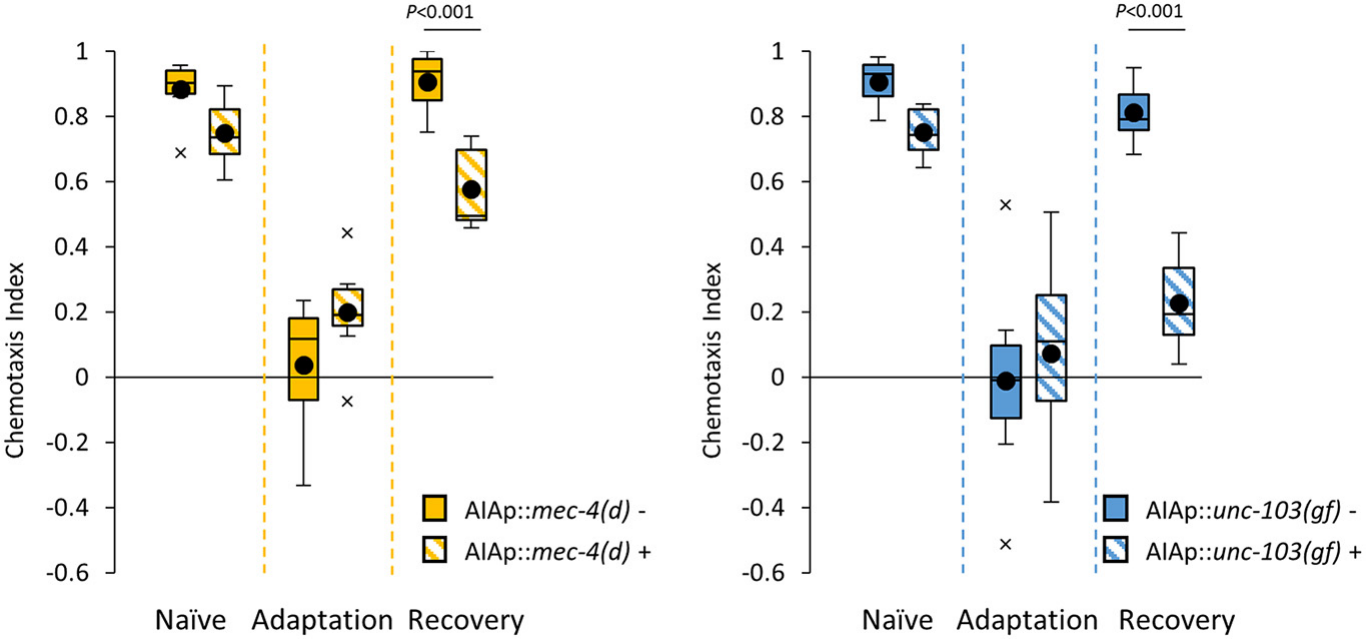
Chemotaxis to AWC-sensed isoamyl alcohol in AIA^–^ animals. Chemotaxis to isoamyl alcohol was analyzed in two AIA^–^ transgenic animals, AIAp::*mec-4(d)* and AIAp::*unc-103 (gf)*, in naive, adapted, and 4 h recovery phases. Boxes, First to third quartiles (Q1 25th to Q3 75th percentile) of each dataset; black line in the boxes, medians; black dots, mean; whiskers, minimum and maximum, excluding outliers (beyond 1.5-fold interquartile range from Q3 and Q1); x, outliers (*n *=* *8, two-way ANOVA, *F*_strain(3,82)_ = 11.4853, *p* = 2.31*e*^−6^, *η^2^* = 0.2959)^h^. *Post hoc t* test with Bonferroni’s correction.

We found that AIA interneurons are required to accelerate forgetting of olfactory adaptation (Figs. 2, 6). Without functional AIAs, conditioned animals were unable to regain chemoattraction toward diacetyl after cultivation with food for 4 h. However, after cultivation with food for 24 h, chemoattraction was recovered in AIA^–^ animals, suggesting that, even in the absence of the functional AIA interneurons, animals can slowly forget. Therefore, AIAs are important to accelerate forgetting of olfactory adaptation.

### AIA interneurons are indispensable for chemotactic behavior to diacetyl only after recovery and, thereby, for behavioral forgetting

**Figure 7.**
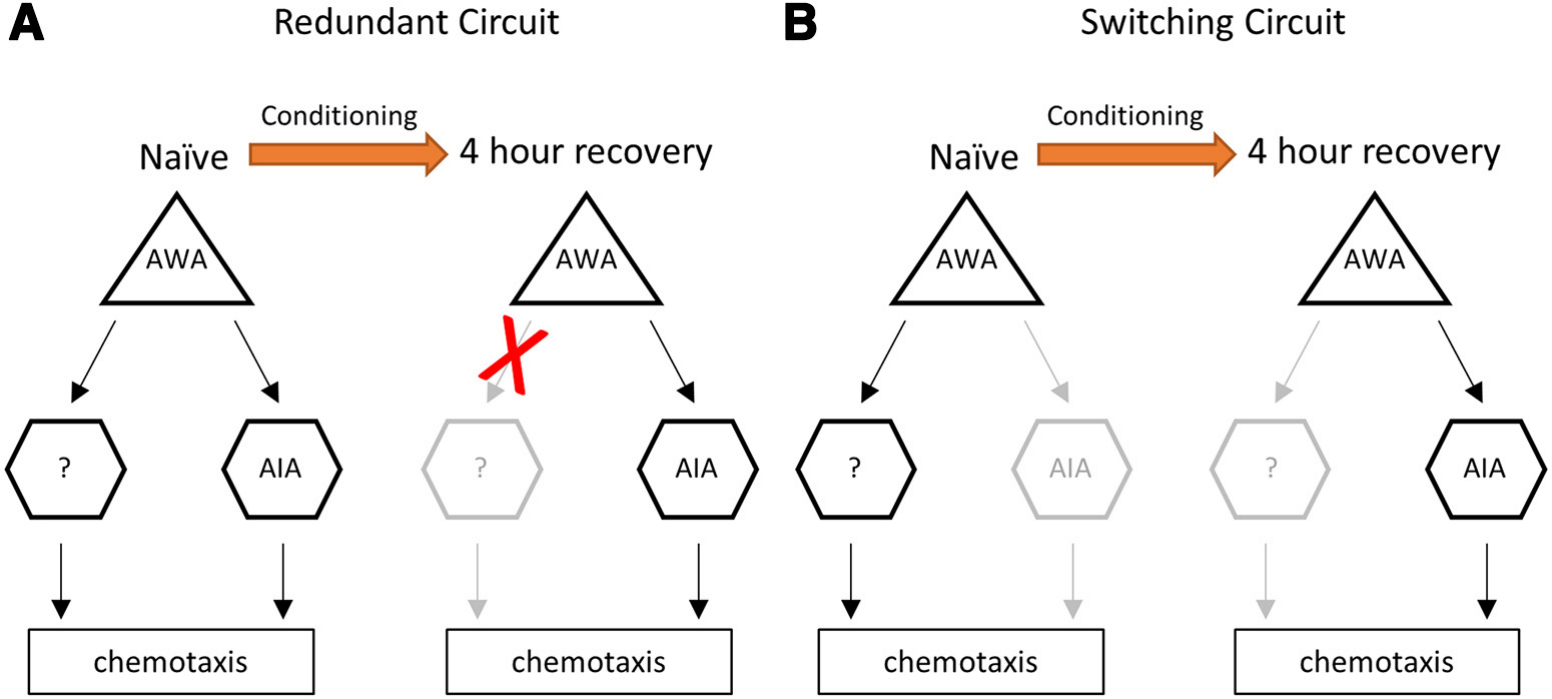
AIAs may regulate forgetting of diacetyl olfactory adaptation via circuit plasticity. ***A***, ***B***, Two hypothetical neural circuits of AIA-dependent behavioral plasticity in the forgetting of olfactory adaptation to diacetyl. In the two models, naive chemotactic behavior might be regulated along with (***A***) or independent from (***B***) an AIA-dependent functional neural circuit. In both models, after conditioning, the AIA-dependent functional neural circuit is required to regulate the corresponding behavioral output after the animal recovers from adaptation.

AIA interneurons are part of the olfactory sensory circuit (Larsch et al., 2015; Dobosiewicz et al., 2019). We observed a minor defect in chemoattraction of naive AIA^–^ animals to diacetyl (Fig. 1*D*,*E*), indicating that the neuronal circuit for chemotaxis can function in naive animals in the absence of AIA interneurons. However, after recovery for 4 h, AIA^–^ animals still showed a defect in chemotaxis to diacetyl (Fig. 1*D*), observed as a defect in behavioral forgetting, although the sensory memory trace declined normally in AWA sensory neurons (Fig. 3). These observations suggest that, in AIA^–^ animals, the sensory response of AWAs cannot induce attractive chemotaxis to diacetyl after conditioning. These results raise two possibilities. One is that although redundant neuronal circuits can regulate chemotaxis to diacetyl in naive animals, after conditioning, the circuit that does not include AIAs becomes nonfunctional so that the AIAs become indispensable for the chemotaxis (Fig. 7*A*). Another one is that, only after conditioning does the neuronal circuit for chemotaxis to diacetyl require the activity of AIAs, which is distinct from the naive circuit (Fig. 7*B*). In these hypotheses, the functional neuronal circuit that does not include AIAs may recover slowly so that chemotaxis to diacetyl recovers after conditioning for 24 h. Our Ca^2+^ imaging analyses of AIA might support the model for the redundant neuronal circuits in naive animals (Fig. 7*A*) because AIA responses are similar to those after the recovery (Fig. 4). These kinds of circuit plasticity, which are based on internal states, are important for behavioral plasticity in higher organisms (Herry et al., 2008; Ramaswami, 2014; Joshua Kim et al., 2017; Kuchibhotla et al., 2017). Furthermore, we suspect that such circuit plasticity involving AIA interneurons might also be used by other olfactory adaptation mechanisms because we also observed that AIA^–^ animals displayed a defective forgetting phenotype toward AWC-sensed isoamyl alcohol without causing a severe chemotactic defect (Fig. 6). To clarify the precise role of AIA interneurons in both circuit and behavior plasticity, additional experiments including optogenetic inactivation or activation of the olfactory circuits in naive and conditioned animals are required to examine these hypotheses.

Our genetic epistasis experiment indicates that AIAs function downstream of the TIR-1 pathway in the regulation of forgetting (Fig. 5). TIR-1 is required to accelerate the forgetting of olfactory adaptation of diacetyl; therefore, the adaptation defect to diacetyl in the *tir-1(gf)* mutant might be caused by forced chemotactic recovery from adaptation during conditioning (Inoue et al., 2013). If this is the case, the suppression of the adaptation defect by AIA^–^ is consistent with the role of AIAs in chemotaxis during the recovery phase.

Our study shows that AIA interneurons in *C. elegans* are required to regulate behavioral forgetting of olfactory adaptations. This indicates that intact neural circuits are important for simple forgetting regardless of the simplicity of the neural system. Studies that reveal learning, memory formation, and forgetting pathways in invertebrates might be conserved across species (Stein and Murphy, 2014; Vorster and Born, 2015; Lipina et al., 2016; Costa et al., 2020; Rahmani and Chew, 2021); therefore, we believe that studies in invertebrates are important to elucidate the mechanisms of forgetting in higher organisms.

## Synthesis

Reviewing Editor: Arianna Maffei, Stony Brook University Decisions are customarily a result of the Reviewing Editor and the peer reviewers coming together and discussing their recommendations until a consensus is reached. When revisions are invited, a fact-based synthesis statement explaining their decision and outlining what is needed to prepare a revision will be listed below. The following reviewer(s) agreed to reveal their identity: Sreekanth Chalasani.

The reviewers agree that the study provide a potential advancement to our understanding of the mechanisms of forgetting. However, significant concerns were raised regarding the solidity of the main hypothesis, as the data supporting it are limited. Additional experiments testing more in depth the effect of AIA on behavior possibly using manipulations of AIA would help solidify the authors’ conclusions.

Reviewer #1

In this paper, the authors show that AIA accelerates the forgetting of olfactory adaptation to diacetyl. Earlier work has shown that calcium levels in AWA are altered in a tir-1-dependent manner to reflect behavior; that is, calcium levels in AWA decrease when the animals lose their attraction to diacetyl right after training, and calcium levels increase again once the animals forget this adaptation and become attracted to diacetyl again. In this paper, ablating AIA or reducing its activity results in slower forgetting as shown by a persistent decrease in attraction to diacetyl after a period of recovery. However, reducing AIA activity does not alter calcium levels in AWC relative to wild-type animals in the different stages of learning/recovery. Additionally, reducing AIA function in a tir-1 gain-of-function background results in the AIA- phenotype rather than the tir-1 gf phenotype. The authors propose that AIA functions in forgetting downstream of the tir-1/AWC-mediated effects and affects behavior independently of calcium changes in AWC. They propose two possible circuits: a redundant circuit where chemotaxis is controlled by both AIA and another unknown neuron but after training and recovery chemotaxis depends on AIA only, or a switching circuit where normally AIA does not participate in the chemotaxis circuit but after training and recovery the circuit switches to depend on AIA.

Overall, this paper presents an interesting possible role for AIA in chemotaxis in a trained animal, but not in an untrained animal. However, the much of the data presented in the paper show how AIA does not work, and not much data shows how AIA does work.

Major revisions:

1. Figure 6 demonstrates two possible mechanisms for AIA’s involvement in chemotaxis in naïve and recovered animals. However, the authors do not really present any evidence to support either of these circuits.

2. Maybe the authors could consider manipulating AIA calcium levels independent of the behavioral paradigm and test if that would have a role in forgetting.

3. In many of the figures, it is unclear why certain pairwise comparisons are being made, but others are not. For example, in Figure 1B, only the gray recovery bars are being compared to WT. In Figure 1C, the blue and gray bars are being compared within strains but the gray bars are being compared across strains.

4. In Figure 2, it seems like the WT data isn’t being compared to the AIA- or tir-1 data at all - but it might be important to show that all three conditions are the same at 24 hours, for example. Since the number of comparisons will affect significance level using Bonferroni’s correction, it is important that the comparisons are consistent across similar experiments (i.e. comparing conditions within strains and/or across strains).

Reviewer #2

In the current manuscript the authors investigate the neural mechanism of forgetting. They give evidence that forgetting is mediated by the AIA neuron downstream of AWA/AWC amphid sensory neurons. The manuscript is well written and follows a clear logic. The experiments support well the claim of the authors.

Minor corrections:

1., Instead of bar graphs, violin or box-plots would be more adequate to visualize data. Also exact significance values instead of stars would be more appropriate.

2., Figure 3B naive data shows no variability (error bars).

3., lane 37: ...C. elegans(Cook... space missing

lane 65: ..., Musashi. (Hadziselimovic.... delete full stop

lane 84, 86, 88, 89, 90: Please correct nomenclature of the Ex lines by adding laboratory codes

lane 151: In tir-1 (tm3036 null)... Correct to tir-1 (tm3036) null

## References

[B1] Bargmann CI (2006) Chemosensation in *C. elegans*. WormBook 2006:1–29. 10.1895/wormbook.1.123.1 PMC4781564 18050433

[B2] Bargmann CI, Hartwieg E, Horvitz HR (1993) Odorant-selective genes and neurons mediate olfaction in C. elegans. Cell 74:515–527. 10.1016/0092-8674(93)80053-H 8348618

[B3] Berry JA, Cervantes-Sandoval I, Nicholas EP, Davis RL (2012) Dopamine is required for learning and forgetting in Drosophila. Neuron 74:530–542. 10.1016/j.neuron.2012.04.007 22578504 PMC4083655

[B4] Berry JA, Cervantes-Sandoval I, Chakraborty M, Davis RL (2015) Sleep facilitates memory by blocking dopamine neuron-mediated forgetting. Cell 161:1656–1667. 10.1016/j.cell.2015.05.027 26073942 PMC4671826

[B5] Berry JA, Phan A, Davis RL (2018) Dopamine neurons mediate learning and forgetting through bidirectional modulation of a memory trace. Cell Rep 25:651–662.e5. 10.1016/j.celrep.2018.09.051 30332645 PMC6239218

[B6] Brenner S (1974) The genetics of Caenorhabditis elegans. Genetics 77:71–94. 10.1093/genetics/77.1.71 4366476 PMC1213120

[B7] Cervantes-Sandoval I, Chakraborty M, MacMullen C, Davis RL (2016) Scribble scaffolds a signalosome for active forgetting. Neuron 90:1230–1242. 10.1016/j.neuron.2016.05.010 27263975 PMC4926877

[B8] Chalasani SH, Chronis N, Tsunozaki M, Gray JM, Ramot D, Goodman MB, Bargmann CI (2007) Dissecting a circuit for olfactory behaviour in Caenorhabditis elegans. Nature 450:63–70. 10.1038/nature06292 17972877

[B9] Chalasani SH, Kato S, Albrecht DR, Nakagawa T, Abbott LF, Bargmann CI (2010) Neuropeptide feedback modifies odor-evoked dynamics in Caenorhabditis elegans olfactory neurons. Nat Neurosci 13:615–621. 10.1038/nn.2526 20364145 PMC2937567

[B10] Cho CE, Brueggemann C, L’Etoile ND, Bargmann CI (2016) Parallel encoding of sensory history and behavioral preference during *Caenorhabditis elegans* olfactory learning. Elife 5:e14000. 10.7554/eLife.14000 27383131 PMC4935464

[B11] Chuang CF, Bargmann CI (2005) A Toll-interleukin 1 repeat protein at the synapse specifies asymmetric odorant receptor expression via ASK1 MAPKKK signaling. Genes Dev 19:270–281. 10.1101/gad.1276505 15625192 PMC545892

[B12] Colbert HA, Bargmann CI (1995) Odorant-specific adaptation pathways generate olfactory plasticity in C. elegans. Neuron 14:803–812. 10.1016/0896-6273(95)90224-4 7718242

[B13] Colbert HA, Smith TL, Bargmann CI (1997) OSM-9, a novel protein with structural similarity to channels, is required for olfaction, mechanosensation, and olfactory adaptation in *Caenorhabditis elegans*. J Neurosci 17:8259–8269. 9334401 10.1523/JNEUROSCI.17-21-08259.1997 PMC6573730

[B14] Cook SJ, Jarrell TA, Brittin CA, Wang Y, Bloniarz AE, Yakovlev MA, Nguyen KCQ, Tang LTH, Bayer EA, Duerr JS, Bülow HE, Hobert O, Hall DH, Emmons SW (2019) Whole-animal connectomes of both Caenorhabditis elegans sexes. Nature 571:63–71. 10.1038/s41586-019-1352-7 31270481 PMC6889226

[B15] Costa JF, Dines M, Lamprecht R (2020) The role of Rac GTPase in dendritic spine morphogenesis and memory. Front Synaptic Neurosci 12:12. 32362820 10.3389/fnsyn.2020.00012 PMC7182350

[B16] Davis RL, Zhong Y (2017) The biology of forgetting—a perspective. Neuron 95:490–503. 10.1016/j.neuron.2017.05.039 28772119 PMC5657245

[B17] Dobosiewicz M, Liu Q, Bargmann CI (2019) Reliability of an interneuron response depends on an integrated sensory state. Elife 8:e50566. 10.7554/eLife.50566 31718773 PMC6894930

[B18] Hadziselimovic N, Vukojevic V, Peter F, Milnik A, Fastenrath M, Fenyves BG, Hieber P, Demougin P, Vogler C, De Quervain DJF, Papassotiropoulos A, Stetak A (2014) Forgetting is regulated via musashi-mediated translational control of the Arp2/3 complex. Cell 156:1153–1166. 10.1016/j.cell.2014.01.054 24630719

[B19] Harbinder S, Tavernarakis N, Herndon L. a, Kinnell M, Xu SQ, Fire A, Driscoll M (1997) Genetically targeted cell disruption in Caenorhabditis elegans. Proc Natl Acad Sci U|S|A 94:13128–13133. 10.1073/pnas.94.24.13128 9371811 PMC24274

[B20] Hardt O, Nader K, Nadel L (2013) Decay happens: the role of active forgetting in memory. Trends Cogn Sci 17:111–120. 10.1016/j.tics.2013.01.001 23369831

[B21] Herry C, Ciocchi S, Senn V, Demmou L, Müller C, Lüthi A (2008) Switching on and off fear by distinct neuronal circuits. Nature 454:600–606. 10.1038/nature07166 18615015

[B22] Inoue A, Sawatari E, Hisamoto N, Kitazono T, Teramoto T, Fujiwara M, Matsumoto K, Ishihara T (2013) Forgetting in C. elegans is accelerated by neuronal communication via the TIR-1/JNK-1 pathway. Cell Rep 3:808–819. 10.1016/j.celrep.2013.02.019 23523351

[B23] Ishihara T, Iino Y, Mohri A, Mori I, Gengyo-Ando K, Mitani S, Katsura I (2002) HEN-1, a secretory protein with an LDL receptor motif, regulates sensory integration and learning in Caenorhabditis elegans. Cell 109:639–649. 10.1016/s0092-8674(02)00748-1 12062106

[B24] Joshua Kim A, Zhang X, Muralidhar S, LeBlanc SA, Tonegawa S (2017) Basolateral to central amygdala neural circuits for appetitive behaviors. Neuron 93:1464–1479.e5. 10.1016/j.neuron.2017.02.034 28334609 PMC5480398

[B25] Kitazono T, Hara-Kuge S, Matsuda O, Inoue A, Fujiwara M, Ishihara T (2017) Multiple signaling pathways coordinately regulate forgetting of olfactory adaptation through control of sensory responses in *C. elegans*. J Neurosci 37:10240–10251. 10.1523/JNEUROSCI.0031-17.2017 28924007 PMC6596540

[B26] Kuchibhotla KV, Gill JV, Lindsay GW, Papadoyannis ES, Field RE, Sten TAH, Miller KD, Froemke RC (2017) Parallel processing by cortical inhibition enables context-dependent behavior. Nat Neurosci 20:62–71. 10.1038/nn.4436 27798631 PMC5191967

[B27] Larsch J, Flavell SW, Liu Q, Gordus A, Albrecht DR, Bargmann CI (2015) A circuit for gradient climbing in C. elegans chemotaxis. Cell Rep 12:1748–1760. 10.1016/j.celrep.2015.08.032 26365196 PMC5045890

[B28] L’Etoile ND, Bargmann CI (2000) Olfaction and odor discrimination are mediated by the C. elegans guanylyl cyclase ODR-1. Neuron 25:575–586. 10.1016/S0896-6273(00)81061-2 10774726

[B29] Lipina TV, Prasad T, Yokomaku D, Luo L, Connor SA, Kawabe H, Wang YT, Brose N, Roder JC, Craig AM (2016) Cognitive deficits in calsyntenin-2-deficient mice associated with reduced GABAergic transmission. Neuropsychopharmacology 41:802–810. 10.1038/npp.2015.206 26171716 PMC4707826

[B30] Rahmani A, Chew YL (2021) Investigating the molecular mechanisms of learning and memory using Caenorhabditis elegans. J Neurochem 159:417–451. 10.1111/jnc.15510 34528252

[B31] Ramaswami M (2014) Network plasticity in adaptive filtering and behavioral habituation. Neuron 82:1216–1229. 10.1016/j.neuron.2014.04.035 24945768

[B32] Rankin CH, Beck CD, Chiba CM (1990) Caenorhabditis elegans: a new model system for the study of learning and memory. Behav Brain Res 37:89–92. 10.1016/0166-4328(90)90074-O 2310497

[B33] Saeki S, Yamamoto M, Iino Y (2001) Plasticity of chemotaxis revealed by paired presentation of a chemoattractant and starvation in the nematode Caenorhabditis elegans. J Exp Biol 204:1757–1764. 10.1242/jeb.204.10.1757 11316496

[B34] Satoh Y, Sato H, Kunitomo H, Fei X, Hashimoto K, Iino Y (2014) Regulation of experience-dependent bidirectional chemotaxis by a neural circuit switch in *Caenorhabditis elegans*. J Neurosci 34:15631–15637. 10.1523/JNEUROSCI.1757-14.2014 25411491 PMC6608435

[B35] Sengupta P, Colbert HA, Bargmann CI (1994) The C. elegans gene odr-7 encodes an olfactory-specific member of the nuclear receptor superfamily. Cell 79:971–980. 10.1016/0092-8674(94)90028-0 8001144

[B36] Sengupta P, Chou JH, Bargmann CI (1996) odr-10 Encodes a seven transmembrane domain olfactory receptor required for responses to the odorant diacetyl. Cell 84:899–909. 10.1016/S0092-8674(00)81068-5 8601313

[B37] Shinkai Y, Yamamoto Y, Fujiwara M, Tabata T, Murayama T, Hirotsu T, Ikeda DD, Tsunozaki M, Iino Y, Bargmann CI, Katsura I, Ishihara T (2011) Behavioral choice between conflicting alternatives is regulated by a receptor guanylyl cyclase, GCY-28, and a receptor tyrosine kinase, SCD-2, in AIA interneurons of *Caenorhabditis elegans*. J Neurosci 31:3007–3015. 10.1523/JNEUROSCI.4691-10.2011 21414922 PMC6623760

[B38] Shuai Y, Lu B, Hu Y, Wang L, Sun K, Zhong Y (2010) Forgetting is regulated through Rac activity in Drosophila. Cell 140:579–589. 10.1016/j.cell.2009.12.044 20178749

[B39] Stein GM, Murphy CT (2014) C. elegans positive olfactory associative memory is a molecularly conserved behavioral paradigm. Neurobiol Learn Mem 115:86–94. 10.1016/j.nlm.2014.07.011 25108196 PMC4250358

[B40] Tomioka M, Adachi T, Suzuki H, Kunitomo H, Schafer WR, Iino Y (2006) The insulin/PI 3-kinase pathway regulates salt chemotaxis learning in Caenorhabditis elegans. Neuron 51:613–625. 10.1016/j.neuron.2006.07.024 16950159

[B41] Vorster AP, Born J (2015) Sleep and memory in mammals, birds and invertebrates. Neurosci Biobehav Rev 50:103–119. 10.1016/j.neubiorev.2014.09.020 25305058

[B42] White JG, Southgate E, Thomson JN, Brenner S (1986) The structure of the nervous system of the nematode Caenorhabditis elegans. Philos Trans R Soc Lond B Biol Sci 314:1–340. 10.1098/rstb.1986.0056 22462104

[B43] Wolfe GS, Tong VW, Povse E, Merritt DM, Stegeman GW, Flibotte S, van der Kooy D (2019) A receptor tyrosine kinase plays separate roles in sensory integration and associative learning in *C. elegans*. eNeuro 6:ENEURO.0244-18.2019–12. 10.1523/ENEURO.0244-18.2019 PMC6712205 31371455

